# Attitudes on Aging Well Among Older African Americans and Whites in South Carolina

**Published:** 2009-09-15

**Authors:** Sara J Corwin, James N Laditka, Sarah B Laditka, Sara Wilcox, Rui Liu

**Affiliations:** Arnold School of Public Health, University of South Carolina; University of North Carolina at Charlotte, Charlotte, North Carolina; University of North Carolina at Charlotte, Charlotte, North Carolina; University of South Carolina, Columbia, South Carolina; University of South Carolina, Columbia, South Carolina

## Abstract

**Introduction:**

Cognitive impairment in older adults is a major cause of functional disability. Interest in protecting brain health is likely to grow as the US population ages and more people have experiences with cognitive decline. Recent scientific evidence suggests that physical activity, heart-healthy diets, and social involvement may help to maintain brain health. We investigated attitudes about aging well among older African Americans and whites to inform the development of interventions to promote cognitive health.

**Methods:**

We used a purposive sample to conduct 5 focus groups with African Americans (n = 42) and 4 with whites (n = 41). Participants also completed a brief survey. In discussions centered on brain health, participants were asked to describe someone they know who is aging well. We used a grounded theory approach to guide the analysis and interpretation of the data.

**Results:**

Both African Americans and whites said that components of aging well include social activity, a strong spiritual life, not taking medications, and traveling. African Americans said aging well means being cognitively intact, free of serious mobility impairment or other health problems, and independent. Whites described aging well as living a long time, staying physically active, maintaining a positive outlook, and having good genes.

**Conclusion:**

African Americans did not commonly associate physical activity with aging well, which suggests that tailored intervention strategies for promoting brain health should emphasize physical activity. African Americans and whites did not commonly associate nutrition with aging well, which also suggests a useful focus for public health interventions.

## Introduction

In the United States, approximately 5 million people have Alzheimer disease. This number is projected to rise to between 11 million and 16 million by 2050 (1,2). Cognitive impairment in older adults is a major cause of functional disability. The public's interest in protecting brain health is likely to grow as the US population ages and more people have experiences with cognitive decline, Alzheimer disease, and related disorders. Public demand for information about maintaining brain health is also likely to be fueled by knowledge that relatively simple health behaviors may help to prevent cognitive decline (3).

Views about successful aging have been extensively examined (4). Research in this area may be categorized into 3 areas of emphasis. First, people who are aging successfully are generally free of disease, cognitively intact, and socially engaged (5). Second, positive spirituality is highlighted (6). Third, aging can be viewed as a dynamic, lifelong adaptive process (7). Empirical studies suggest that successful aging includes characteristics from all 3 theories (4,8-11). Recent work has also suggested that leisure activities contribute to successful aging (12). Although useful, many of these studies rely on  small samples (8,9,12) or homogeneous groups, such as older Canadian men (4), residents of the Netherlands (11), or Japanese Americans in Washington State (10).

Much less is understood about perceptions of successful aging in the context of maintaining cognitive health or about racial or cultural variations in these perceptions. Understanding such differences is relevant because of the increasing racial and cultural diversity of the US population. The prevalence, incidence, and cumulative risk of Alzheimer disease may be considerably higher among African Americans than among whites (1). Given recent evidence that healthy lifestyles may reduce the risk of cognitive decline, Alzheimer disease, and vascular dementia (3,13), effective health promotion strategies must be developed. Understanding how diverse groups view cognitive impairment and the health behaviors that have been linked with its prevention is a useful first step.

Our study uses qualitative data from the Healthy Brain Project, an initiative of the Prevention Research Centers Healthy Aging Research Network (PRC HAN), funded by the Centers for Disease Control and Prevention (CDC) (14,15). The goal of the Healthy Brain Project is to establish a science base that will support public health interventions to promote brain health in diverse populations, including populations that may differ by region (16). Using focus group data, we examined attitudes about aging well in the context of maintaining cognitive health. The objectives of our study were to 1) examine attitudes about aging well among older community residents in South Carolina, 2) explore similarities and differences between perceptions of aging well among African Americans and whites, and 3) suggest ideas for health promotion strategies.

## Methods

### Recruitment

From November 2005 through March 2006, we conducted 9 focus groups with community residents aged 50 years or older in South Carolina. The discussion guide was pilot tested with 1 group of older African Americans to ensure that the questions were clear and appropriately sequenced. Although limited funding precluded more extensive pilot testing, results suggest that participants understood the questions and that the questions elicited useful information. Most focus groups with white participants were conducted at community senior centers, whereas most African American groups were convened at area churches. Contacts at each site recruited English-speaking adults who self-reported minimal or no cognitive impairment. The age criterion is consistent with CDC's definition of older adults, particularly regarding interventions designed to affect the older life course (14,15).

### Procedures

Before each focus group, in accordance with the University of South Carolina institutional review board policies, participants signed informed consent forms. A 9-item focus group discussion guide, developed collaboratively for use across all HAN sites, was designed to elicit participants' awareness and attitudes about several topics related to brain health, brain disorders, and the behaviors associated with maintaining brain health (Table 1). We report the findings from the first question: "Without mentioning a name, please tell us about someone who you think is aging well." We report only the results for 1 question in this study because the results are exclusive to South Carolina, and the results of other questions are reported elsewhere as part of the larger, multisite study (16-18). African American moderators and assistants conducted the African American focus groups. White moderators and assistants conducted the white focus groups. The focus groups lasted 90 to 120 minutes. During the audio-recorded sessions, the moderators used probes to obtain and clarify responses (19). Participants also completed a brief paper-and-pencil survey that included demographic questions and questions about selected health behaviors and mental health characteristics. Participants received a $30 gift card for their time.

### Data analysis

A professional service transcribed the audio recordings into Microsoft Word (Microsoft Corporation, Redmond, Washington). To develop a codebook, we randomly selected 1 transcript each from the African American and white focus groups. Using the discussion guide as an initial framework, 2 researchers independently read and manually marked each text segment in both transcripts that expressed a unique idea or meaning. The same 2 researchers, during an open coding process (20), reached consensus about the meaning of each code (intercoder agreement 0.80). These 2 researchers read and coded the remaining 7 transcripts while a third researcher created the codebook and entered the manually coded data. New codes were added to the electronic codebook as they were identified.

Transcripts were imported into ATLAS.ti version 5.0 (ATLAS.ti Scientific Software Development, Cologne, Germany) for coding and analysis. A data matrix was generated to tabulate code frequencies within and by group. The tabulations were examined for links to other codes. This axial coding process (20) connected code categories and identified relationships representing common themes. We compared and contrasted themes within and across racial and ethnic groups (a process known as the "constant comparison method") (21) to identify similarities and differences in the data.

The data collected from the participant survey (n = 83) were entered into EpiInfo version 6.0 (Centers for Disease Control and Prevention, Atlanta, Georgia). SAS version 9.1.3 (SAS Institute, Inc, Cary, North Carolina) was used to analyze the survey data and to compare sociodemographic and other characteristics by race. Continuous variables were analyzed with Student *t* tests, categorical variables with χ^2 ^tests.

## Results

### Demographic characteristics

The results presented are from 83 respondents (42 African American, 41 white) who participated in 1 of 9 focus groups (5 African American, 4 white). Compared with white participants, African American participants were nearly 10 years younger, had significantly higher body mass index (BMI), and were less likely to report limiting saturated fat intake (Table 2). Whites were more likely to report at least 30 minutes of moderately intense physical activity 5 days per week and thus fulfilled a widely accepted guideline for physical activity (22). Other characteristics did not differ significantly by race.

### Focus group themes

Both African Americans and whites expressed the belief that aging well included being socially involved, having an active spiritual life, not taking medications, and traveling for leisure (Table 3).

In nearly every group, participants discussed how "being involved with one another" is essential not only for "keeping yourself occupied"  but for health benefits as well. One white participant explained that social relationships are a "mind relief."  Another elaborated, "If you are interested in other people and what they do, you sorta forget yourself along the way."  An African American participant explained how many things, including social engagement, contributed to her mother's aging well:

My mother was old but I don't think she never got bored cause she used to visit people and walk 4 and 5 miles just to visit and come back. Go fishing. And she was in her right mind when she died. At 89 she was in her right mind. All that kept her active.

Others described the importance of laughter in social settings as a way to "stay young." For example, a white participant said, "Laughter's very good for you. At this age . . . if you don't laugh, you're going to be crying."

Regardless of race, many participants said that having an active spiritual life was vital for aging well. Many cited the importance of prayer and expressing gratitude to God: "I thank the Lord for my health and strength . . . . I do," and "You've got to thank God first." A white participant summarized a view expressed by both African Americans and whites that faith was important:

And I think my faith in God keeps me going. You know, it's comforting when things go wrong or a little bit of things get out of balance. I've always got Him to depend on and my faith keeps me going.

Although whites and African Americans were equally likely to express the value of an active spiritual life, African Americans, more than whites, mentioned church as part of an active spiritual life. Several African Americans said that being a "faithful church member," "going to church," and "leading Bible study" were important for aging well.

Participants of both races frequently commented that not taking medications was an indication of aging well. Participants did not distinguish between over-the-counter and prescription medications; however, they were clear in citing their absence as an aspect of aging well. The following were typical responses: "And I'll be 72 . . . and I don't take no medicine," and "I'm the only one in my graduating class that don't take high blood pressure medication."

Traveling for leisure was a strong theme that emerged from all of the focus groups. Many participants noted that they "travel a lot" and "travel more now than [they] ever did." Several explained that social aspects of traveling contributed to aging well "because it gets you out of the house . . . you interact with other people." Others mentioned that they enjoyed organized "group trips," and they were "always busy doing things and going places."

Differences between African Americans and whites in perceptions of aging well were evident in several areas (Table 3). Whites more frequently described the following as characteristics of aging well: living a long time, staying physically active, having a positive attitude, and inheriting "good" genes. African Americans more often mentioned that aging well involved having no cognitive impairment, being physically mobile, maintaining independence, and experiencing few or no health problems. African Americans and whites also identified different types of leisure activities that contribute to aging well.

While a few African Americans noted that a person's age is an indicator of aging well, whites mentioned this phenomenon much more often. Repeatedly, white participants described a person who was aging well in terms of chronological age ("she's 99 and 7 months," "she's in her 80s," "she'll be 90 in May"). Many participants were quick to reveal their own age as an example of aging well: "I'm 71, and I have just taken up golf."

Whites discussed physical activity and exercise considerably more often than did African Americans. Furthermore, whites much more commonly noted that aging well involved the ability to exercise or engage in physical activity apart from leisure activities. The following are specific examples from whites: "It's a little more involved than just sitting at home and trying to exercise," "She's healthy and does jumping jacks," and "She does everything . . . exercises us all down!" The few African Americans who mentioned physical activity relative to aging well did so in the context of "getting around" and being free of serious physical impairment.

A dimension of aging well mentioned only by whites was having a positive "attitude" or good "mental outlook." One participant stated, "I think your mental outlook has an awful lot to do with it [aging well] . . . Most of these people around here [community center] are very young at heart." Another participant felt that a person's psychological state was connected to other dimensions of health: "If you have a good mental outlook, you're probably going to be active and you're probably going to eat reasonably well." Other participants noted that "not feeling sorry for yourself" and having a "very young attitude" are also essential for aging well.

Only white participants noted that genetics played a role in aging well. Several said that "it all comes from heredity" and "heredity is the name of the game." Often, white participants shared that their parents' longevity and health conditions predisposed them for the same: "If your parents got a heart trouble, chances are you gonna have it." The following dialogue further illustrates this theme:

Participant 1: My dad was 100 and my mother was 102.

Participant 2: No wonder you are at your age now.

Participant 3: That's why she's so good.

Participant 2: That's why she's good . . . you got it on both sides.

Although a few white participants discussed being "sharp" or "clear minded" as an aspect of aging well, the theme appeared often in the African American groups. Participants often characterized this by referring to people who could "remember things that happened a long time ago," and who had "nothing wrong" with their minds. Other African Americans noted that being oriented was a sign of aging well: "Someone who doesn't forget . . . they can tell you what day it is," and "what's going on with daily events."

Although the importance of being independent was mentioned by participants of both races, this theme was expressed notably more often in the African American groups. For example, physical mobility was frequently used to describe aging well: "She gets around better than the average teenager," and "I just get around real good." Driving was very commonly mentioned as a characteristic of aging well. One African American stated with pride: "I can drive and carry myself wherever I want to go."

Another aspect related to the theme of maintaining independence mentioned with particular emphasis by African Americans was the importance of continuing to work. Work was described a variety of ways, including volunteering ("she delivers Meals on Wheels"), housework, and part-time or full-time employment. One African American described how continuing to work contributes to physical well-being and functioning:

You know, I talked to a guy yesterday and he's in his early 70s and you may think this is far-fetched, but this guy said, "I have never been tired." He goes and he still works. He does everything. He's sort of a jack-of-all-trades. He's always doing something . . . always doing something. He said he just can't sit around. He said, "People get off work and say: 'I'm tired.' " He said, "I've never been tired!"

Finally, "living independently" and being "independent" were aspects of the theme of physical functioning as a characteristic of aging well.

The absence of health or medical problems was noted as an attribute of aging well only by African Americans. Several said that being in "good health" meant not "having any health problems," and not going "to the doctor but for a checkup once a year."

Participants of both races cited leisure activities as an important aspect of aging well. There were, however, differences by race in the types of activities mentioned. Whites more commonly mentioned dancing ("line dancing," "square dancing," "swing") and music ("I'm 80 and I play in a band and I shake at both ends"). African Americans more commonly noted that arts and crafts were important for aging well, specifically mentioning sewing, quilting, and painting.

## Discussion

Our study compared perceptions about aging well among older African Americans and whites in 1 region of South Carolina, in the context of maintaining cognitive health. No studies have examined aging well specifically in the context of cognitive health, as viewed by people in South Carolina, where health behaviors increase the vascular damage that elevate the risk of cognitive decline. Furthermore, little research has focused on the differences in views about aging well between African Americans and whites. We noted similarities in views about aging well among African Americans and whites. Focus group participants of both races said that important aspects of aging well included social activity, a strong spiritual life, not taking medications, and traveling and other leisure activities. The findings suggest that, for older adults, the concept of aging well is multidimensional (4,8-11). They also reinforce the importance of spirituality in aging well (4,6) and the connection between leisure and successful aging (12).

Differences in attitudes by race also emerged. African Americans said aging well meant being cognitively intact, physically mobile, independent, and free from health problems. Whites described aging well in terms of living a long time, staying physically active, maintaining a positive outlook, and having good genes.

Limitations to this study should be considered. Our sample was a purposive sample of mostly older women drawn from 1 area in South Carolina. African Americans were primarily recruited through churches, whereas whites were recruited through senior centers. This recruitment difference may account for some of the differences by group. Other limitations include the use of self-reported measures for body mass index, levels of physical activity, perceptions of stress, location of residence, and quality of memory. Participant responses may have been inaccurate or biased by a desire to comment in socially desirable ways. Finally, despite the best efforts of highly trained focus group moderators, some participants may speak more than others and may influence others' opinions or their willingness to speak.

Recent scientific evidence suggests that healthy behaviors improve one's chances of maintaining cognitive and emotional health (3). White older adults in our study discussed staying physically active as a way to age well; for this population, it may be useful to emphasize the connection between physical activity and cognitive health, specifically, to provide an additional motivation to become physically active. African Americans did not discuss physical activity as a component of aging well. For this population, it may be useful to motivate physical activity by emphasizing that brain health is a prerequisite of participating actively in the church and Bible study, of being "sharp" or "clear minded" and having a good memory, of avoiding involvement in the health care system, and of living independently and continuing to travel, drive, work, and volunteer. The connection between physical activity and remaining independent may be a particularly motivating message for African Americans, who valued independence.

Whites emphasized the role of genetics in aging well. For this group, it may be useful to communicate the likelihood that genetic predispositions are often moderated by behaviors. For example, evidence suggests that physical activity and heart-healthy diets may reduce the risk of brain disorders (22-24). African Americans and whites did not commonly associate nutrition with aging well, which also suggests a useful focus for health promotion interventions. Health promotion and prevention strategies could also focus on the shared perceptions (23) of aging well. For example, health communications focused on social involvement and spirituality may be meaningful to both African Americans and whites.

## Figures and Tables

**Table 1:** Focus Group Discussion Questions and Prompts Used in the Healthy Brain Project^a^

Question/Item	
1.	Without mentioning a name, please tell us about someone who you think is aging well. Prompt: Describe characteristics and/or qualities.
2.	What words do you use to describe seniors/older people who *do* have a loss of memory or thinking ability? Prompt: Or, *do not* have a loss of memory or thinking ability?
3.	Tell us about any concerns you may have about your ability to keep your memory or ability to think as you age. Prompt: Importance of? Why important?
4.	Describe the things that we can do to keep our brains healthy and keep our memories or our ability to think as we age.
5.	What sort of things have your heard from TV, radio, newspapers or magazines, the Internet and so forth about keeping your brain healthy?
6.	Introduction of the concept of brain health prevention through proper diet, maintaining a healthy weight, being physically active, and being socially involved. Participants are then asked: What do you think/feel about what I just said (read)? Prompt: What changes regarding your diet, activity level, or social involvement are you willing to make?
7.	What do you think would be the most effective ways to motivate you, or other people, to keep their brains healthy in terms of diet, physical activity, and social involvement?
8.	If we put you in charge of getting the word out to others about the importance of a healthy diet, physical activity, and being socially involved for healthy brains, what would you do to make sure everyone knew about it? Prompt: Specific examples of promotional materials. Prompt: Types of messages or slogans? Prompt: Where to place materials and messages?
9.	Is there anything else that you would like to talk about?

a The Healthy Brain Project is a multisite initiative of the Prevention Research Centers Healthy Aging Research Network, funded by the Centers for Disease Control and Prevention' Healthy Aging Program (14,15).

**Table 2:** Characteristics of Participants in Focus Group on Aging Well, by Race, South Carolina, 2005-2006

Variable	African American (n = 42)	White (n = 41)	*P* Value^a^
Age, mean (SD), y	67.3 (7.9)	76.8 (7.9)	<.001
BMI, mean (SD), kg/m^2^	32.1 (9.3)	27.2 (4.4)	.01
Stress^b^, mean (SD), days/week	4.8 (8.3)	4.5 (7.3)	.69
**Sex, %**			
Female	82.1 **c**	87.8	.47
**Marital status, %**			
Married	40.5	32.5	.45
Not married **d**	59.5	67.5	
**Highest education completed, % **			
<High school	26.2	27.5	.76
High school graduate or GED	33.3	27.5	
Some college, technical or vocational	28.6	25.0	
≥College degree	11.9	20.0	
**Annual income, $^e^ **			
<20,000	52.8	51.5	.48
20,000-39,999	33.3	24.2	
≥40,000	13.9	24.2	
**Social activity, %**			
Very social	36.6	47.5 **f**	.17
Somewhat social	56.1	52.5 **f**	
Not very social	7.3	0.0 **f**	
**Meets PA recommendations**	9.5	26.8	.04
**Memory, %**			
Fair or poor	15.0	22.5	.39
Good, very good, excellent	85.0	77.5	
**Saturated fat, %**			
Limiting fat currently	63.4	87.5^f^	.01
Thinking about limiting fat	36.6	10.0^f^	
Do not plan to limit fat	0.0	2.5^f^	
**Residence^g^**			
Rural	41.5	60.0	.09
Urban	58.5	40.0	

Abbreviations: BMI, body mass index; GED, general educational development; PA, physical activity (22).

a For continuous variables, the nonparametric Kruskal-Wallis test was used; pairwise comparisons used the Kolmogorov-Smirnov test, comparing African Americans with whites. For categorical variables, χ^2^ tests were performed.

b Stress was defined as the self-reported number of days in the past 30 that the participant felt "a lot of stress, depressed, anxious, or had problems with their emotions."

c Because of missing data in this category, n = 39 for African American participants.

d "Not married" includes single, separated, divorced, or widowed.

e Because of missing data in this category, n = 36 for African American participants; n = 33 for white participants.

f Because of missing data in this category, n = 40 for white participants.

g Self-reported location of residence. Because of missing data in this category, n = 40 for white participants.

**Table 3:** Themes Regarding Aging Well, by Race, From Focus Groups With Older African American and White Adults, South Carolina, 2005-2006

Theme	Responses		

	Both (9 Focus Groups)	African American (5 Focus Groups, 42 Participants)	White (4 Focus Groups, 41 Participants)
Social life	Social involvement Socially active	NA	NA
Spiritual life, religiosity, and organized religion	Active spiritual life Prayer Gratitude	Faithful church member Going to church Bible study	NA
Mental health	Not worrying	Being "sharp" or "clear minded" Oriented to daily events Good memory	Positive attitude Good mental outlook Humor
Physical health	Not taking medications	Few health problems See doctors only for checkups	Genetics Heredity Living to advanced age (longevity)
Leisure activities	Travel	Arts and crafts	Dancing, music
Physical activities	NA	Basic mobility (eg, being able to walk, "getting around")	Exercise Physical activity Staying active
Independence	NA	Living independently Driving Working (eg, volunteering, housework, employment)	NA NA NA

Abbreviation: NA, not applicable (participants did not mention the theme).
